# Stimulus complexity and retelling output in aphasia: an exploratory single-case analysis

**DOI:** 10.3389/fnhum.2026.1679562

**Published:** 2026-04-13

**Authors:** Hyunsoo Yoo

**Affiliations:** Department of Communication Science and Disorders, Robbins College of Health and Human Science, Baylor University, Waco, TX, United States

**Keywords:** aphasia, discourse profiling, narrative discourse, story retelling, structural complexity

## Abstract

Discourse-based aphasia therapies often rely on narrative stimuli, yet the structural characteristics of these materials remain largely unexamined. While stimulus complexity may influence language production and cognitive load, few studies have proposed quantifiable methods for profiling discourse-level structure. This study developed and applied a transparent, rule-based structural profiling approach for narrative stimuli used in story-retelling therapy. Twelve stimuli were analyzed using a Python-based pipeline to extract sentence count, average sentence length, complex sentence ratio, and a surface-level segmentation proxy (Estimated Information Units). Composite scores were calculated using z-score normalization and used to classify stimuli into Low, Medium, and High complexity groups. As a proof-of-concept application, produced Information Unit (IU) change was examined descriptively to contextualize stimulus-level patterns across treated versus evaluation-only conditions. Stimuli varied in structural complexity, and IU change patterns were variable across stimuli. These findings demonstrate the feasibility of quantifying structural properties of narrative stimuli and illustrate how stimulus profiling can support stimulus calibration and sequencing in discourse-based aphasia research. Further work with larger samples is needed to validate these observations and evaluate broader applications of structural profiling in discourse-level intervention.

## 1 Introduction

In conversational contexts, the ability to listen, comprehend, and rephrase a partner’s message plays a central role in functional communication (Clark and Brennan, 1991; Glosser and Deser, 1992; Kagan and Simmons-Mackie, 2007). This process involves more than passive reception of information; it engages integrated cognitive-linguistic operations including listening, comprehension, reconstruction, and verbal output (Ulatowska et al., 1983). At the heart of this ability is story retelling, which supports essential discourse functions such as appropriate response, topic maintenance, and contextual alignment (Marini et al., 2005).

Unlike spontaneous narrative production, story retelling places heavy demands on verbal working memory, as it requires the temporary storage and reorganization of aurally presented linguistic input. Yoo and McNeil (2019) demonstrated that in individuals with aphasia, performance on story retelling tasks is significantly correlated with measures of verbal working memory (*r* = 0.50–0.75). When story retelling abilities are impaired due to aphasia, individuals often struggle to follow conversational turns and maintain contextual coherence, which can negatively impact not only the efficiency of daily interaction but also broader aspects of social participation and relationship-building.

Story retelling is more than a sequence of sentence repetitions. It requires the integration of narrative structure, temporal sequencing, and causal coherence, placing complex demands on both linguistic and cognitive processing resources (Stein and Glenn, 1979; Trabasso and van den Broek, 1985). This task is often conceptualized as narrative retelling in the discourse literature, where it is regarded as a discourse-level function commonly used in both assessment and treatment contexts (Liles, 1993).

A range of studies has identified four categories of factors influencing story retelling performance. First, cognitive factors such as working memory capacity and processing speed have been shown to significantly influence discourse-level comprehension and organization. For example, See and Ryan (1995) demonstrated that individual differences in these cognitive capacities mediate age-related variations in discourse processing performance. From a theoretical perspective, discourse comprehension models (e.g., Van Dijk and Kintsch, 1983; Kintsch, 1988) highlight the central role of working memory in supporting macrostructural integration, coherence building, and the real-time coordination of incoming and stored information across discourse segments.

Second, linguistic factors contribute to discourse production through several interrelated mechanisms. According to Levelt (1989), lexical retrieval involves a multi-stage process that includes conceptual preparation, lemma selection, and phonological encoding. When any part of this process is disrupted, speakers may experience breakdowns in fluency and lexical cohesion, which can negatively impact the continuity of narrative expression. Syntactic comprehension also plays an essential role, particularly when processing structurally complex sentences such as those with embedded clauses or noncanonical word orders. Gibson (1998) Syntactic Prediction Locality Theory explains that increased syntactic complexity elevates both integration and memory costs, which can interfere with the real-time construction and maintenance of coherent discourse. Building on this account, expectation-based models of sentence processing suggest that the difficulty of comprehension is influenced by how well upcoming structures align with probabilistic syntactic expectations (Levy, 2008). The use of cohesive devices is equally important for sustaining both local and global coherence across utterances. Proper use of linguistic markers such as pronouns, conjunctions, and lexical ties helps maintain the semantic flow of discourse. In a study comparing children with and without language disorders, Liles (1993) reported that limited or inappropriate use of cohesive devices was associated with poorer narrative organization and reduced listener comprehension. Collectively, findings on lexical retrieval, syntactic comprehension, and cohesion highlight the critical role of linguistic processing in discourse production and demonstrate that deficits in any of these domains may compromise performance at the discourse level.

Third, discourse-level factors also play a critical role in narrative performance, particularly through schematic knowledge and causal event representation. Mandler and Johnson (1977) first demonstrated that narrative comprehension and recall are guided by underlying story grammar structures. Building on this foundation, Stein and Glenn (1979) proposed that a well-formed narrative is organized around an internalized story schema, typically consisting of elements such as setting, initiating event, goal, attempt, and outcome. This mental framework guides both comprehension and production by helping individuals structure events in a temporally and causally coherent sequence. Marini et al. (2005) found that individuals with language impairments often struggle to construct causal relationships between events, resulting in fragmented or poorly integrated narratives. These findings highlight the importance of macrostructural organization in discourse-level processing.

Fourth, stimulus-level characteristics have also been shown to influence language performance, particularly through structural complexity and modality of presentation. Thompson and Shapiro (2005), in their work on Treatment of Underlying Forms (TUF), demonstrated that training on syntactically complex sentences led to generalization to simpler, untrained structures. Although this work was conducted at the sentence level, it underscores a key principle observed in the Treatment of Underlying Forms (TUF) literature: training on structurally complex sentence structures is associated with generalization to linguistically related, simpler, untrained structures (Thompson and Shapiro, 2005). While not focused on discourse per se, these findings provide conceptual justification for considering stimulus complexity as an active design element in discourse-based aphasia therapy.

However, despite the growing recognition of stimulus-level effects, structural complexity has rarely been examined in discourse-based intervention. While structural complexity at the sentence level has been well studied, particularly through treatment approaches such as Treatment of Underlying Forms (TUF), which shows that training on complex syntax can promote generalization (Thompson and Shapiro, 2005), this principle has seldom been extended to the discourse level. Discourse-based therapies continue to rely heavily on narrative stimuli, and while clinicians may informally consider aspects of narrative structure such as temporal ordering and causal relationships, stimuli are typically selected based on topic familiarity, cultural relevance, or perceived appropriateness for the client (Dipper et al., 2020). Structural characteristics of the stimuli, including syntactic complexity or information density, are rarely quantified or systematically manipulated as experimental variables. This lack of quantification and calibration of discourse-level structure represents a methodological gap that limits both empirical validation and stimulus design in current aphasia therapy.

This framework builds on prior findings but shifts the analytic focus from output to input. To address this gap, the present study introduces a quantifiable method for structural profiling of narrative stimuli used in aphasia intervention. This method uses four linguistic indicators for this purpose: sentence count, average sentence length, complex sentence ratio, and estimated information units. We applied a Python-based pipeline (Python Software Foundation, 2023) to analyze 12 story stimuli. Composite complexity scores were then computed via z-score normalization and used to classify stimuli into low, medium, and high complexity tiers.

These four structural indicators were selected because they represent complementary dimensions of narrative complexity identified in prior discourse and psycholinguistic research. Sentence count reflects discourse length and may index overall informational load (Van Dijk and Kintsch, 1983); average sentence length indexes syntactic load and processing demands associated with longer or more syntactically dense clauses (Just and Carpenter, 1992). The complex sentence ratio captures clause embedding and hierarchical structure, which are central markers of structural complexity in aphasia and sentence-processing research (Thompson and Shapiro, 2005). Estimated Information Units provide a surface-level approximation of information density by indexing clause-like segmentation cues (Halliday and Hasan, 1976; Nicholas and Brookshire, 1993). Together, these indicators provide a structural profile of the linguistic demands imposed by each narrative, distinct from extralinguistic influences such as topic familiarity, emotional salience, or cognitive state, which were beyond the scope of this study.

As a proof-of-concept application, we explored how this profiling framework might be used to characterize stimuli and inform stimulus characterization and calibration in future research. By shifting analytic focus from narrative output to stimulus input, this study offers a preliminary demonstration of data-informed stimulus profiling for potential use in discourse therapy.

## 2 Method

### 2.1 Design

This study employed a single-case pre-post design to (1) quantify the structural complexity of narrative stimuli and (2) descriptively examine how stimulus-level structural complexity relates to produced IU change in a proof-of-concept application within story retelling therapy (SRT). The study consisted of two components: a structural analysis of 12 narrative stimuli and an intervention phase. Structural features including sentence count, average sentence length, complex sentence ratio, and estimated information units were extracted using a Python-based pipeline. *Z*-score normalization was applied, and composite structural complexity scores were calculated and used to group stimuli into Low, Medium, and High Load Blocks based on tertile distribution.

### 2.2 Participants

A single participant took part in this study: a right-handed, monolingual English-speaking woman, aged 70 at the time of participation. Her educational background included 17 years of formal schooling, and she had previously worked in a professional administrative role at a university before retirement. Approximately 34 months prior to the study, she experienced a left hemisphere stroke that was diagnosed by a neurologist in 2022. Aphasia was confirmed for the purposes of this study using Western Aphasia Battery-Revised (WAB-R) during the pre-treatment assessment. Based on the WAB-R scores, she presented with mild anomic aphasia (AQ = 92.7). She did not exhibit signs of apraxia of speech. All procedures were approved by the Institutional Review Board (IRB) at Baylor University, and informed consent was obtained prior to participation.

### 2.3 Procedure

#### 2.3.1 Structural feature extraction and stimulus analysis

To quantify stimulus-level complexity, four structural features were analyzed for each of the 12 story stimuli. These structural features were selected not only for their theoretical relevance to syntactic and propositional complexity, but also for their practical utility in automated discourse analysis. The selection of these four structural features was guided by four key criteria:

1. conceptual grounding in prior research on syntactic load and information density,
2. extractability using standard Python-based string processing methods [e.g., str.split(), str.count(), re.search()],
3. objectivity and consistency through rule-based implementation, and
4. applicability across all 12 stimuli to support reliable comparison.

This approach was intended to support the calculation of a composite score that would be both theoretically interpretable and computationally reproducible. All features were extracted using the same explicit Python rules across all stimuli to support reproducibility.

All computations were conducted in Python 3.11 (Python Software Foundation, 2023) using standard libraries including pandas (McKinney, 2010), numpy (Harris et al., 2020), and re (Python Software Foundation, 2023) for data processing, and matplotlib (Hunter, 2007) for visualization. Linguistic features were extracted using basic string handling methods in Python 3.11, including str.split() for word segmentation, len() for word count calculation, and str.count() for detecting conjunctions and punctuation-based information units. All subsequent calculations, normalization, and block assignment were performed using pandas, numpy, and re.

1. Sentence Count

This measure was defined as the total number of syntactically complete sentences per stimulus, determined through manual segmentation based on independent clause boundaries rather than punctuation alone. Initial segmentation was estimated using regular expression-based punctuation splits, but final sentence counts were manually corrected to reflect functional sentence boundaries and clause completeness.

1. Complex Sentence Ratio

This measure was defined as the proportion of sentences containing at least one subordinating conjunction (e.g., because, although, when, if, since, though, unless, while). Each sentence was flagged as complex if it included any of these conjunctions, detected using regular expression searches.


$$\begin{array}{l} \text{Complex Sentence Ratio}\\ =\frac{\begin{array}{l} \;\text{Total Sentence CountNumber of sentences}\\ \;\;\;\;\;\;\;\;\;\;\;\text{with subordinating conjunctions} \end{array}}{\text{Total Sentence Count}} \end{array}$$

1. Average Sentence Length

This measure was defined as the mean number of words per sentence calculated by dividing the total word count by the manually verified sentence count. Word counts were extracted using Python’s string splitting and length functions.


Average Sentence Length=Total Word Count/Sentence Count

1. Estimated Information Units (IUs)

This measure was defined as a proxy for informational density, estimated as the sum of all commas, occurrences of ‘and’, and occurrences of ‘but’ in each stimulus. These elements were treated as proxies for clause-level segmentation and cohesion markers. This operationalization was chosen because it offers a simple and reproducible indicator of clause-like segmentation in short narrative texts, which was essential for the structural profiling goal of this study.


$$\text{Estimated}\;\mathrm{IUs}=\text{count of commas}+{\text{count of}}^{"}{\text{and}}^{"}+{\text{count of}}^{"}{\mathrm{but}}^{"}$$

Estimated IUs provide a surface-level approximation of informational density within the written stimulus and are not directly comparable to produced IUs derived from spoken retellings. Accordingly, Estimated IUs were used solely for stimulus-level structural profiling and not as a standardized production-based IU metric. Because punctuation marks such as commas can be inconsistently applied, a subset of the narrative stimuli was screened prior to analysis to check obvious punctuation irregularities. Given the limited scope of this screening, punctuation-based segmentation was treated as a surface-level approximation for stimulus-level profiling (e.g., Halliday and Hasan, 1976; Graesser et al., 2004; McNamara et al., 2014; Taboada, 2019).

1. Normalization and Composite Score

Raw values for each feature were z-score normalized to allow for cross-feature comparison. A composite structural load score was calculated for each stimulus by averaging the four z-scores. The composite score was used as a practical index to rank stimuli for block assignment. Because different feature profiles can yield similar composite values, we report feature-level measures alongside the composite score (Table 1) to support interpretation. Each raw feature was converted to a z-score, where x represents the raw score for a given stimulus, *μ* is the mean, and *σ* represents the standard deviation across all stimuli:


$$z=\frac{x-\mu }{\sigma }$$

**Table 1:** Structural analysis of narrative stimuli.

Story	Sentence count	Word count	Avg sentence length	Complex sentence ratio	Estimated IUs	Composite score	Load block
Airport	14	220	15.71	0.50	21	0.698	High
Gas*	14	217	15.50	0.43	21	0.550	High
Tickets	13	212	16.31	0.85	13	0.513	High
Fire	13	223	17.15	0.54	16	0.474	High
Water	14	220	15.71	0.36	16	0.131	Medium
Paint	14	224	16.00	0.29	16	0.091	Medium
Garage Sale	14	214	15.29	0.43	13	−0.077	Medium
Library*	13	202	15.54	0.62	10	−0.199	Medium
Loan*	16	200	12.50	0.25	16	−0.261	Low
Sandwich	14	202	14.43	0.50	11	−0.304	Low
Tightrope	14	217	15.50	0.14	12	−0.511	Low
Baseball	11	152	13.82	0.45	12	−1.106	Low

Structural features of the 12 narrative stimuli. Sentence count values were manually verified. Composite scores were calculated as the mean of z-scored structural variables (sentence count, average sentence length, complex sentence ratio, and estimated IUs). Load Block assignments were based on tertile split of composite scores. *Evaluation-only stimuli are marked with an asterisk.

The Composite Structural Load Score was then computed for each stimulus as the mean of its four z scores of normalized features: sentence count, complex ratio, sentence length, and estimated IUs:


$$\begin{array}{l} \text{Composite Score}=\\ \;\frac{z\text{Sentence Count}+z\text{Complex Ratio}+z\text{Sentence Length}+z\mathrm{IUs}}{4} \end{array}$$

1. Block Assignment (Load Level)

Stimuli were categorized into Low, Medium, or High Load Blocks based on their Composite Structural Load Scores, using tertile distribution. Specifically, the top third of scores were classified as High Load, the middle third as Medium Load, and the bottom third as Low Load. This classification was implemented using the qcut() function from the Python pandas library.

Although additional linguistic variables (e.g., lexical frequency, cohesion indices, semantic diversity, narrative schema structure) may also influence discourse performance, the present study intentionally limited feature selection to a small set of transparent, rule-based metrics to prioritize reproducibility and methodological clarity in this proof-of-concept profiling framework.

#### 2.3.2 Analytical framework for IU response profiling

In this exploratory analysis, we examined how these structural profiles corresponded to IU gains. Structural complexity scores were drawn directly from the four z-standardized features described above. IU gains were computed as the difference in the number of IUs produced before and after treatment. A total of 12 narrative stimuli (*n* = 12) were included in the analysis: nine were used as active treatment stimuli, and three were used solely for evaluation to assess generalization and retention effects. The nine treated stories were presented within therapy sessions and evaluated using pre-treatment and immediate post-treatment retellings collected within the same session. The three evaluation-only stories were not used in therapy but were retold at three time points: pre-treatment, immediately after completing all treatment sessions (Post1), and 7 weeks post-treatment (Post2). These stimuli were used to assess the generalization and maintenance of treatment gains. IU change gains for each interval (Post1–Pre, Post2–Pre, and Post2–Post1) were then derived to evaluate directional patterns over time.

The relationship between stimulus complexity and IU gain was examined using Pearson correlation analysis and visualized using scatterplots with regression lines. This allowed comparison of changes in IU output between treated and untreated stimuli. Given the limited sample size, the analysis was exploratory in nature and aimed to identify directional trends rather than statistical significance. The correlation analysis was conducted using the scipy.stats.pearsonr() function in Python.

#### 2.3.3 Clinical application: SRT protocol and data collection

To generate the empirical data required for the profiling analysis, the stimuli were implemented within the following SRT protocol. The participant completed nine face-to-face story retelling treatment (SRT) sessions over a three-week period. Each session was conducted by the Principal Investigator (PI) and lasted approximately 1 h. A total of 12 narrative stimuli that underwent structural profiling were used: nine were assigned as treatment stimuli (corresponding to the nine treatment sessions), and three were used exclusively as evaluation-only stimuli. The story stimuli were administered using the Story Retelling Procedure (SRP; McNeil et al., 2001) and were originally adapted from the Discourse Comprehension Test (DCT; Brookshire, 1993), a standardized discourse measure with well-established reliability and stimulus control.

Each treatment session consisted of an auditory presentation of a story stimulus, a pre-treatment retelling, a multimodal therapy phase, and a post-treatment retelling. These components were implemented in a predefined order and were consistent across sessions. All retellings were performed based on auditory input without the presence of visual stimuli during the retelling task itself.

The multimodal therapy phase included repetition of target words and phrases, brief reading and writing activities, picture-sequence and sentence-ordering tasks, true/false comprehension checks, story summarization (beginning–middle–end), and guided retelling at progressively larger units. All retellings were transcribed word-for-word and scored for Information Units (IUs) using a predefined IU framework for each stimulus. The participant was not instructed to retell verbatim; paraphrases were credited when they mapped onto a predefined IU category, and each IU was counted only once per retelling regardless of repetition. IU differences between pre- and post-treatment retellings were used as exploratory outcome measures in the present study.

Across the nine treatment sessions, one narrative stimulus was trained per session in a fixed one-stimulus-per-session schedule. This schedule guaranteed that all nine treatment-assigned stimuli were addressed over the course of the nine sessions. Because this study focuses on structural profiling rather than treatment efficacy, only a brief procedural outline is provided here.

Three formal assessments were conducted for the evaluation-only stimuli: Pre (Test 1), Post1 (Test 2), and Post2 (Test 3). Pre (Test 1) was administered prior to treatment, Post1 (Test 2) immediately after the final treatment session, and Post2 (Test 3), conducted 7 weeks later, assessed only the evaluation-only stimuli. The delay between Test 2 and Test 3 was originally planned as a four-week interval but was extended to 7 weeks due to a fall experienced by the participant. Evaluation-only stimuli were administered at all three assessment timepoints to examine generalization and maintenance. For treated stimuli, pre- and immediate post-treatment retellings were collected within the same therapy session. No delayed post-treatment assessment was conducted for these stimuli. Performance was measured using the number of Information Units (IUs) produced in retellings.

## 3 Results

### 3.1 Stimulus analysis: structural complexity of 12 stories

**Figure 1:**
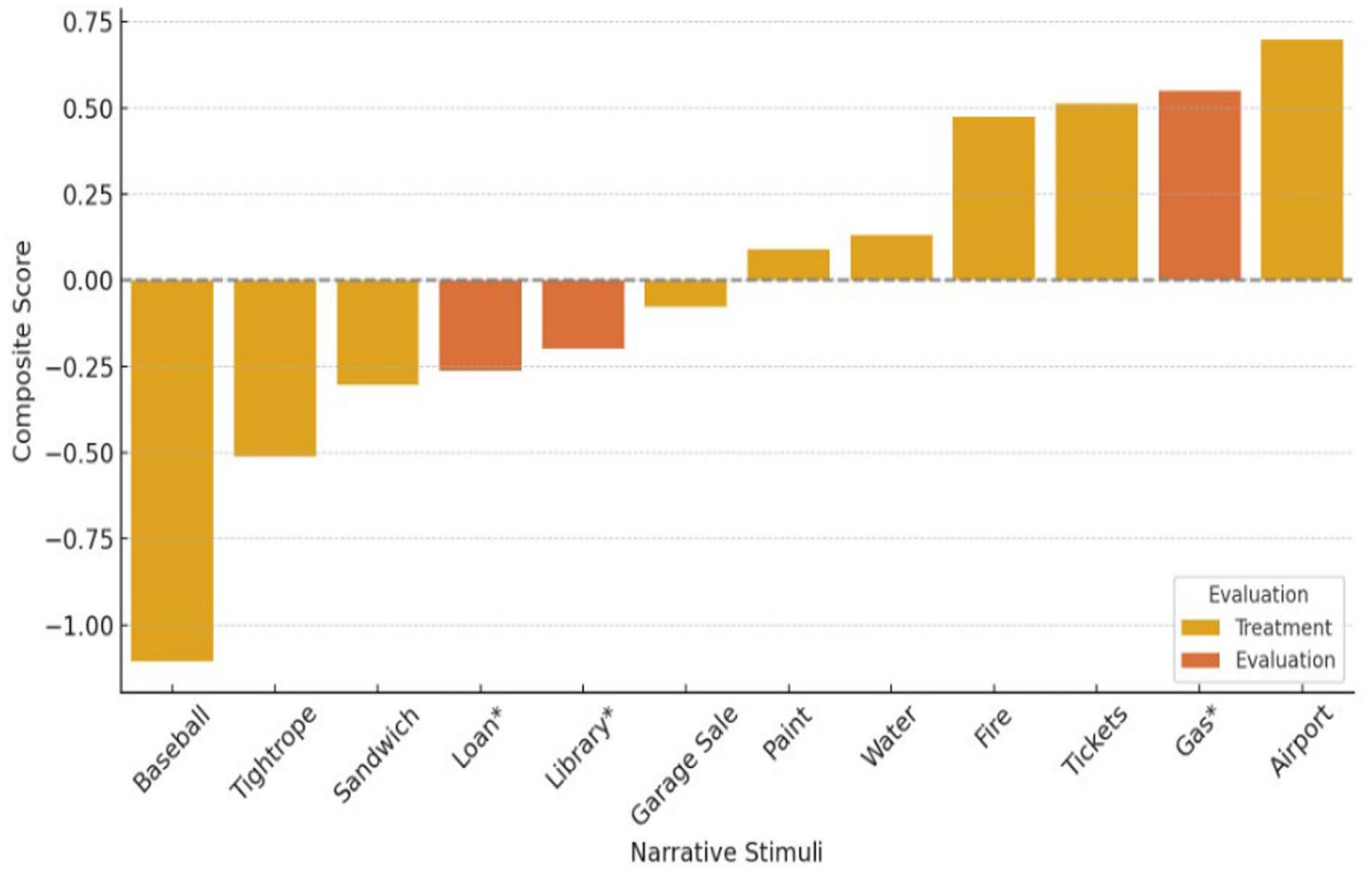
Composite structural complexity scores of 12 stories. Evaluation stimuli are marked with an asterisk (*) and highlighted in a different color.

Stimuli were divided into Low, Medium, and High Load Blocks based on tertile distribution of composite scores (see Table 1; Figure 1). Four stimuli were assigned to each block (High = 4, Medium = 4, Low = 4) permitting exploratory comparisons across tiers.

### 3.2 Exploratory aim: relationship between structural complexity and IU gain

#### 3.2.1 Treatment stimuli

For the nine treatment stimuli, IU gain was calculated as the difference between post-treatment and pre-treatment retellings. In this case, high-load stories such as *Airport* (Composite = +0.70, Gain = +25), *Tickets* (+0.51, +30), and *Fire* (+0.47, +41) were associated with relatively higher IU gains. In contrast, low-load stimuli showed mixed gains (e.g., *Tightrope*: −0.51, +13; *Sandwich*: −0.30, +46).

**Figure 2:**
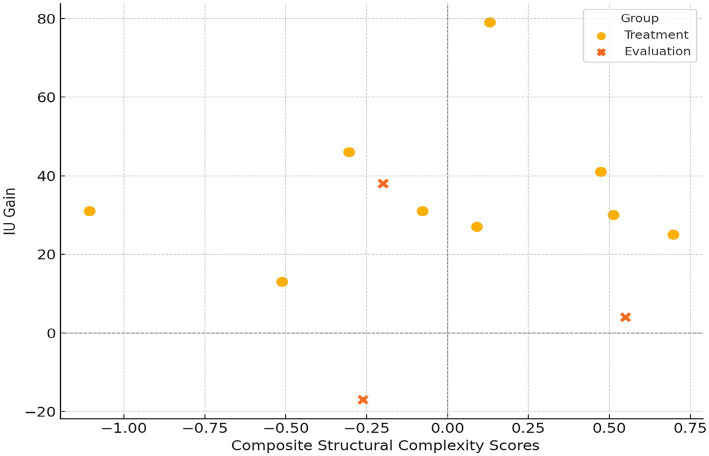
Relationship between composite structural complexity scores and IU gain. Each point represents a stimulus. Treatment stimuli are shown as orange circles and evaluation-only stimuli are shown as red x’s. IU gain for evaluation stimuli is calculated as Post2–Pre.

**Figure 3:**
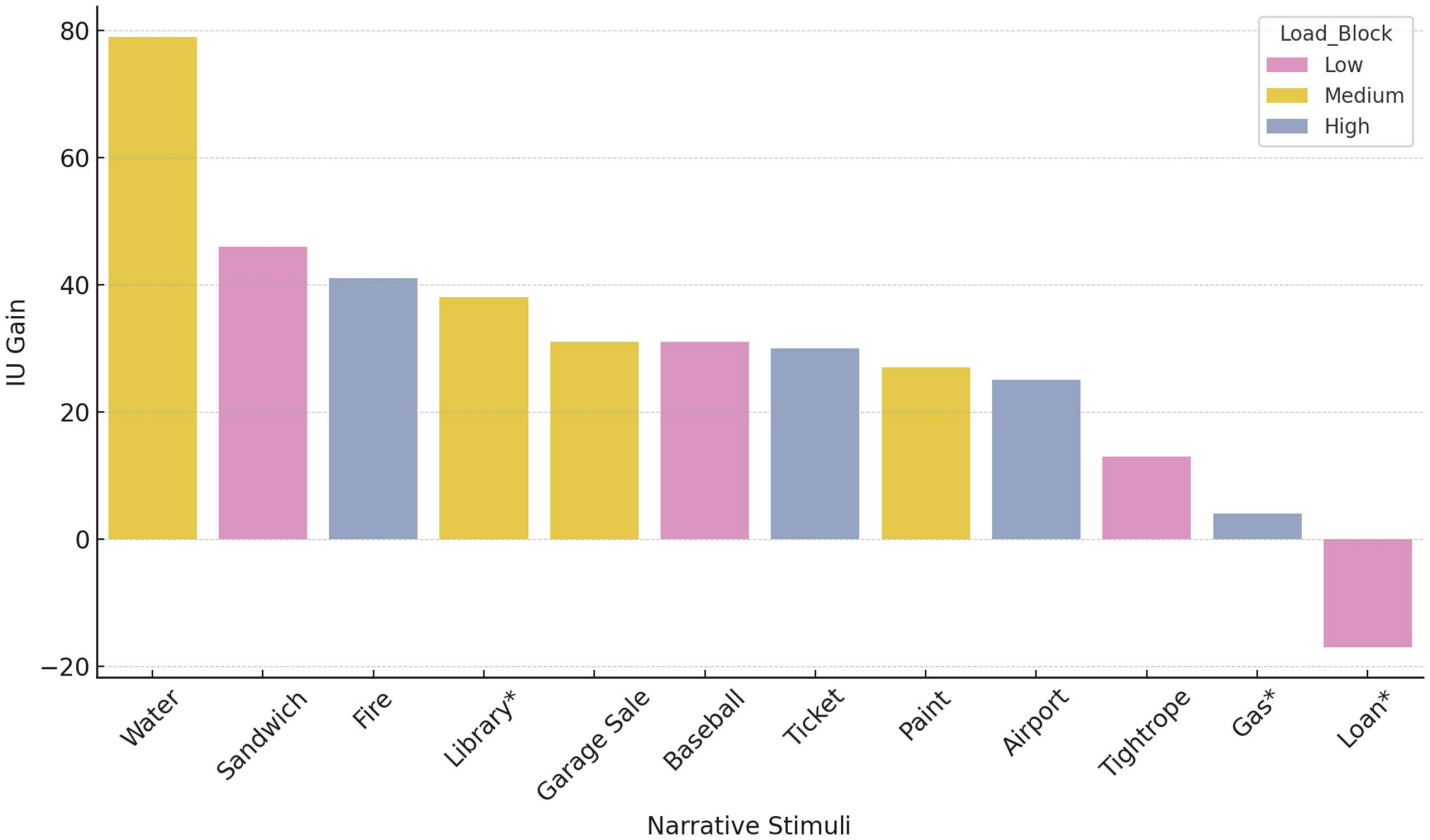
IU gain by narrative stimuli, grouped by structural complexity (Load Block). Colors represent Load Block classification: Low (pink), Medium (yellow), and High (blue). Evaluation-only stimuli are marked with an asterisk (*); IU gain was calculated as the difference between post- and pre-treatment (or Post2–Pre for evaluation-only stimuli).

Table 2 presents the composite complexity scores and corresponding IU gains for all stimuli, while Figures 2, 3 visualize the relationship between complexity and treatment response. The average IU gain for High Block treatment stimuli was +32, compared to +30 for Low Block stories. A very weak positive correlation was observed between composite structural complexity and IU gain (*r* = +0.13); this exploratory result should be interpreted with caution given the small sample size and the context-specific nature of the data.

**Table 2:** Structural profiles of the 12 narrative stimuli, including composite structural load scores and load block assignments.

Story	Composite scores	IU Gain	Load block
Airport	0.698	25	High
Gas*	0.55	4	High
Tickets	0.513	30	High
Fire	0.474	41	High
Water	0.131	79	Medium
Paint	0.091	27	Medium
Garage Sale	−0.077	31	Medium
Library*	−0.199	38	Medium
Loan*	−0.261	−17	Low
Sandwich	−0.304	46	Low
Tightrope	−0.511	13	Low
Baseball	−1.106	31	Low

For treated stimuli, IU gain reflects Post1–Pre; for evaluation-only stimuli (marked with *), IU gain reflects Post2–Pre. IU, Information Unit. Load blocks were assigned based on tertile distributions of composite structural load scores.

### 3.3 Evaluation-only stimuli (*n* = 3)

Among the three evaluation-only stimuli, IU gain was calculated as the difference between Post2 and Pre sessions. The three evaluation-only stimuli represented different load levels (Gas = High, Library = Medium, Loan = Low), and they showed divergent patterns: Loan (Composite = −0.26) yielded a decrease in IU score (−17), while Library (Composite = −0.20) showed the highest gain across all stimuli (+38).

Specifically, the magnitude of IU gain followed the order of Library (Medium), Gas (High), and Loan (Low), with the Medium-load stimulus showing the most pronounced improvement. A correlation analysis suggested no consistent association between structural complexity and IU gain (*r* = −0.07); however, this result should be interpreted with caution given the very small sample size (*n* = 3). The lack of a strong correlation may reflect idiosyncratic participant response patterns or potential floor and ceiling effects within specific narrative items, which can inflate variability and potentially obscure the underlying relationship between stimulus complexity and IU gains.

**Figure 4:**
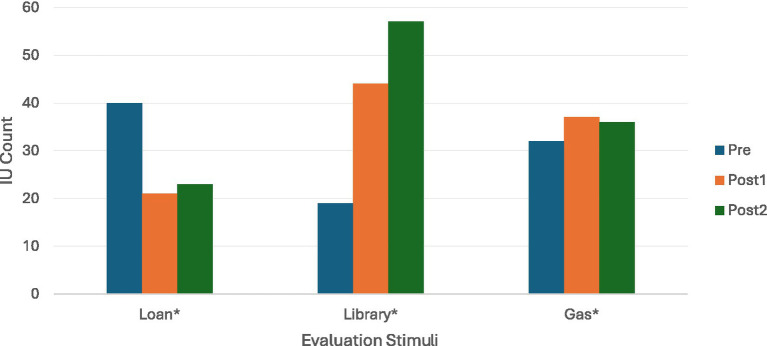
IU count for evaluation-only stimuli across Pre, Post1, and Post2. IU counts across Pre, Post1, and Post2 for evaluation-only stimuli: Loan* (Low load), Library* (Medium load), and Gas* (High load).

In addition to overall IU gains, temporal trajectories for the evaluation-only stimuli were examined across three timepoints: Pre, Post1, and Post2 (Figure 4). *Library* showed an increase from 19 to 44 and then to 57 IUs across the three timepoints. *Gas* remained at 32, 37, and 36 IUs, while *Loan* moved from 40 to 21 and then to 23 IUs. In particular, the steady growth observed in the Medium-load Library stimulus contrasts with the stability of the High-load Gas and the decline in the Low-load Loan stimulus. Taken together, these patterns illustrate a wide range of outcome trajectories across evaluation-only stimuli with different structural loads.

**Figure 5:**
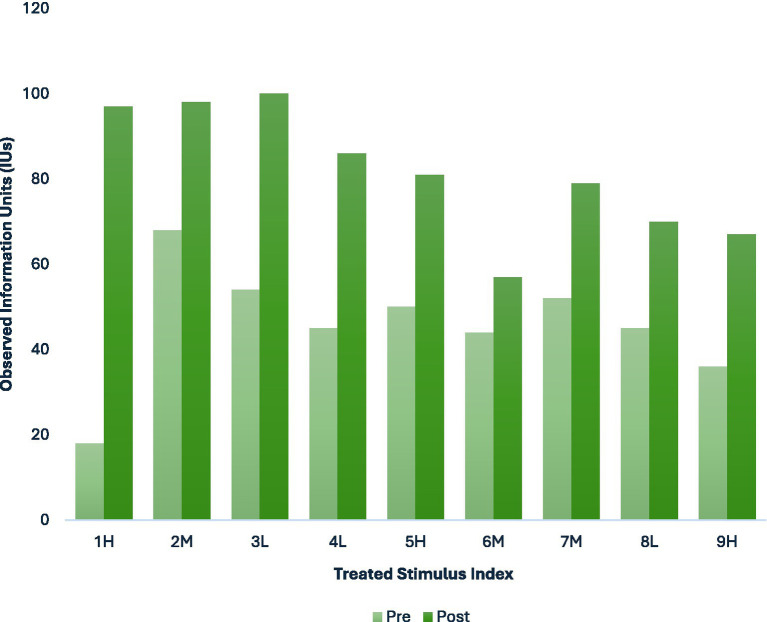
Pre- and post-session IU counts within individual treatment sessions for treated narrative stimuli. Values are provided for descriptive purposes to contextualize stimulus-level gain calculations and are not intended to support inferential claims about treatment efficacy. Stimuli are labeled by load level (L, Low; M, Medium; H, High) based on the composite structural load classification.

Figure 5 presents pre- and post-session Information Unit (IU) counts for each treated narrative stimulus. These values are provided to contextualize the gain scores used in the stimulus-level analyses by illustrating baseline IU levels (i.e., room-for-growth) as well as the range and variability of IU counts across individual treatment sessions. The figure is intended to support interpretation of observed variations rather than to evaluate treatment effectiveness through statistical or inferential analyses.

## 4 Discussion

This study quantitatively analyzed the structural complexity of narrative stimuli used in aphasia therapy and explored how this complexity related to discourse-level treatment responsiveness. While previous studies have focused on features of patient-produced discourse (Brookshire and Nicholas, 1994; Glosser and Deser, 1992), this study uniquely investigated the structural properties of the stimulus materials themselves and examined how those properties interacted with treatment outcomes. Several key findings emerged.

First, the correlation between composite structural complexity scores and IU gain across all 12 stories was minimal (*r* = 0.05), indicating no consistent association. When the analysis was restricted to treated stimuli only (*n* = 9), the association remained weak (*r* = +0.13), highlighting substantial variability in treatment responsiveness across stimuli with similar structural load. Rather than dismissing the role of complexity, it is possible that structural complexity functions as a moderator interacting with other factors, such as auditory comprehension, topic familiarity, task repetition, or individual cognitive profiles (Caplan and Waters, 1999; Daneman and Carpenter, 1980). Such an interaction may account for the observed variability, which the subsequent analysis begins to unpack. Narrative performance may be shaped by factors not directly examined in the present study, including extralinguistic factors such as topic familiarity, emotional salience, cognitive state, and aphasia severity. While these factors lie beyond the scope of the structural metrics examined here, acknowledging their potential influence provides important context for interpreting the observed patterns.

Second, when stimuli were grouped by complexity level (Low, Medium, High), Medium Load stimuli showed higher average IU gains (43.75) than High Load (25.0) and Low Load (18.25) groups. While this descriptive trend emerged from a small number of items per group, this tentative pattern may be interpreted in relation to learning principles described in Cognitive Load Theory (Sweller et al., 2011) and Vygotsky’s Zone of Proximal Development (Vygotsky, 1978), which posit that materials of moderate difficulty can support optimal learning when appropriately matched to the learner’s capabilities. This observation could also be explored in future work in relation to broader models of arousal and performance, such as the Yerkes-Dodson Law (Yerkes and Dodson, 1908).

Third, a general trend toward higher gains in treated stimuli was observed, with substantial variability across items. Treatment stimuli, delivered with repetition, multimodal support, and clinician feedback, led to overall higher IU gains. Evaluation stimuli, by contrast, had a lower average gain (8.33) and less variability across items. Notably, the evaluation-only stimulus *Library* (Composite = −0.20) yielded a substantial IU gain (+38) despite not being directly trained. This pattern may reflect stimulus-specific cognitive or thematic factors. It is also plausible that the stimulus benefited from thematic familiarity or alignment with pre-existing narrative schemas (Bartlett, 1932; Van Dijk and Kintsch, 1983). For example, situations involving libraries often include familiar actions such as reading, borrowing books, or interacting in quiet, structured settings, which are widely shared and reinforced in cultural experience (Nelson, 1986). These schema-consistent elements may have facilitated access to relevant linguistic and conceptual representations during retelling. In addition to such thematic familiarity, it is also plausible that the participant internalized strategies or representations that extended beyond the trained material (Ulatowska et al., 1981). Together, these cognitive advantages, stemming from both familiar content and the potential internalization of discourse strategies, may have jointly contributed to the observed generalization in this stimulus. Accordingly, the observed gain in this case may be associated with cognitive advantages linked to familiar and semantically coherent content. However, because narrative familiarity and participant-specific relevance were not measured or analyzed in this study, this interpretation should be considered speculative.

Taken together, the variability observed in this case may reflect influences beyond structural complexity. It is theoretically possible that multiple factors, including treatment conditions, thematic properties of the stimuli, and individual background knowledge and cognitive state, contributed to the observed changes (Kay and Ellis, 1987; Edmonds et al., 2009). Although similarity between trained and untrained stories may influence performance on evaluation-only items, the present dataset includes only three untrained stimuli, making it impossible to meaningfully examine similarity-based generalization mechanisms. This question represents an important direction for future research using larger stimulus sets and multi-participant samples.

Additionally, because evaluation stimuli were administered without treatment or feedback, they may have more directly reflected the raw cognitive burden imposed by structural complexity. In contrast, the observation that several high-complexity treatment stimuli yielded substantial IU gains suggests that the therapeutic context—through repetition, scaffolding, and feedback—may have enabled the participant to manage or even overcome the processing demands posed by more complex narratives. If substantiated in future studies, this pattern suggests that the therapeutic context, which incorporated repetition and clinician scaffolding, may have enabled the participant to manage the processing demands of more complex narratives (Thompson and Shapiro, 2005; Thompson et al., 2003).

In summary, the findings of the present study demonstrate that structural complexity at the discourse level is a multidimensional construct that can be modified through coordinated adjustments to multiple linguistic features, rather than as a simple binary factor. This multidimensional perspective allows narrative stimuli to be sequenced or adapted not only based on thematic content but also on structural characteristics, providing a foundational framework for the clinical and theoretical implications discussed in the following section.

### 4.1 Clinical relevance and theoretical implications

Taken together, the findings show that structural complexity can be quantified to profile and organize narrative stimuli, and that produced IU change varies across narrative stimuli. Accordingly, structural complexity may be considered as a descriptive feature for characterizing narrative stimuli and interpreting variability in stimulus-level responses. However, further research is needed to clarify how structural complexity interacts with observed variability before it can be reliably used to guide interpretation. A structurally informed approach to stimulus design may support descriptive examination of how input characteristics differ across narrative stimuli. The present approach may be relevant for future exploratory work on stimulus profiling and characterization. In this view, structural complexity can be conceptualized as a measurable descriptive feature that may be examined and compared across narrative stimuli.

## 5 Limitations and future directions

This study has several limitations. First, the single-case design and the use of only 12 stimuli limit both the generalizability and the interpretability of the findings. Given the variability, generalization beyond the present participant cannot be assumed, regardless of aphasia severity or educational background. These constraints also affect the ability to systematically analyze the mechanisms underlying the relationships between structural complexity and IU gains.

Second, while four structural features were quantified, other potentially influential variables were not examined. These include linguistic factors such as lexical sophistication (Kyle and Crossley, 2015), imageability (Paivio, 1991), verb argument structural complexity (Levelt, 1989; Thompson and Shapiro, 2007), and referential cohesion (Halliday and Hasan, 1976), which may affect discourse processing and production in nuanced ways. Additionally, features such as lexical frequency, semantic density, cohesion indices, and narrative schema familiarity were not included in the present profiling model. As these multidimensional variables may interact with structural complexity to influence narrative performance, their omission limits the explanatory power of the current structural profile.

Moreover, external variables such as topic familiarity, emotional salience, and cognitive state at time of testing were not controlled, particularly for evaluation-only stimuli. These factors may contribute to the variability observed across stories with similar complexity scores. Additionally, although the study involved retelling tasks, the range of discourse contexts was relatively limited, primarily reflecting performance under constrained conditions rather than fully naturalistic discourse. Detailed outcome-focused reporting was also beyond the scope of this manuscript, as these aspects are addressed in a separate treatment-focused manuscript^1^.

Future research should incorporate larger samples and more diverse stimulus sets, ideally using model-based approaches to capture interactions among stimulus complexity, participant profiles, and treatment conditions. Integrating real-time discourse data and longitudinal follow-up may offer additional insight into how stimulus features support long-term functional outcomes. Taken together, these limitations underscore the need for multidimensional frameworks that systematically integrate structural, cognitive, and contextual factors in discourse-level aphasia rehabilitation.

## 6 Conclusion

This single-case study demonstrates that the structural complexity of narrative stimuli can be systematically measured and categorized using quantifiable features. However, given the absence of consistent relationships in this small and variable data set, it is possible that structural complexity did not meaningfully influence IU gains in this specific case. Nevertheless, the results demonstrate diverse response trajectories across narrative stimuli, highlighting the feasibility of a reproducible approach to stimulus input calibration within discourse-based therapy research. In particular, the sustained improvement observed in one of the Medium-load stimuli suggests that structural profiling may offer a valuable framework for understanding variability in treatment responsiveness, although this should be interpreted cautiously given the single-case design. Further empirical research is needed to validate these observations and to explore the broader applicability of multidimensional structural profiling in discourse-level intervention.

## Data Availability

The original contributions presented in the study are included in the article/Supplementary material, further inquiries can be directed to the corresponding author.

## Footnotes

^1^Yoo, H. (in press). Story Retelling Therapy for improving spoken discourse in very mild aphasia: A single-case feasibility study. *Aphasiology*.
