# Supplementary material for: Stimulus complexity and retelling output in aphasia: an exploratory single-case analysis
Source: Front Hum Neurosci. 2026 Apr 13;20:1679562. doi: 10.3389/fnhum.2026.1679562 (PMC13113798; doi:10.3389/fnhum.2026.1679562)
Supplement: Supplementary file 1 [file Data_Sheet_1.pdf]

For each narrative stimulus in the dataset:

1. Preprocessing:
   a. Load the raw story text as a single string.
   b. Standardize spacing and remove leading/trailing whitespace.

2. Sentence Segmentation:
   a. Split text into initial sentence candidates using punctuation (".", "!", "?").
   b. Manually verify and adjust segments to reflect independent clause boundaries.
   c. Count the number of syntactically complete sentences → Sentence Count.

3. Complex Sentence Detection:
   a. Define a list of subordinating conjunctions (e.g., "because", "although", "if", "when", "since", "though", "while", "unless").
   b. For each sentence:
      i. Search for any subordinating conjunction.
      ii. If found, classify sentence as complex.
   c. Compute Complex Sentence Ratio = (Number of Complex Sentences) / (Total Sentence Count)

4. Average Sentence Length:
   a. Tokenize text into words (e.g., using whitespace splitting).
   b. Compute total word count.
   c. Average Sentence Length = Total Word Count / Sentence Count

5. Estimated Information Units (IUs):

a. Count the number of commas in the text.

b. Count the number of "and" and "but" occurrences (case-insensitive, surrounded by spaces).

c. Estimated IUs = Commas + "and" + "but" counts

6. Z-score Normalization:

a. For each of the four features (Sentence Count, Complex Ratio, Sentence Length, Estimated IUs):

i. Compute z = (value - mean) / standard deviation, using dataset-wide statistics.

b. Store the normalized z-scores.

7. Composite Structural Complexity Score:

a. Compute the average of the four z-scores.

b. Composite Score = (z1 + z2 + z3 + z4) / 4

8. Load Block Classification:

a. Use tertile cutoffs to divide all stimuli into three Load Blocks:

- Top third → High Complexity

- Middle third → Medium Complexity

- Bottom third → Low Complexity

b. Assign the current stimulus to one of the three blocks based on its Composite Score.

End loop.
